# The Influence of Patient Sex on Outcomes Following One-Stage and Two-Stage Revision for Periprosthetic Joint Infection in Total Joint Arthroplasty

**DOI:** 10.3390/antibiotics12091392

**Published:** 2023-08-31

**Authors:** Rory W. Metcalf, Taylor Rowe, Megan Tersteeg, Malcolm E. Dombrowski, Susan Odum, Jesse E. Otero

**Affiliations:** 1 OrthoCarolina Research Institute, Charlotte, NC 28207, USA; rmetcalf53@siumed.edu (R.W.M.);; 2 Saint Louis University School of Medicine, St. Louis, MO 63104, USA; 3 OrthoCarolina Hip & Knee Center, Charlotte, NC 28207, USA; 4 Department of Orthopaedic Surgery, Atrium Health Musculoskeletal Institute, Charlotte, NC 28207, USA; 5 OrthoCarolina Hip & Knee Center, Atrium Health Musculoskeletal Institute, Charlotte, NC 28207, USA

**Keywords:** periprosthetic joint infection, revision surgery, total knee arthroplasty, total hip arthroplasty

## Abstract

Although females have a higher rate of primary total joint arthroplasty (TJA), males have a higher rate of revision. The literature lacks studies examining the relationship between sex and outcomes following single and two-stage exchange for periprosthetic joint infection (PJI). The purpose of this study was to examine if differences exist in outcomes following revision for chronic PJI between sexes. A retrospective review was performed on all patients with an MSIS confirmed PJI who underwent a single or two-stage exchange at our institution from January 2010 to January 2021. Patient demographics, comorbidity characteristics, and outcomes were collected and compared between males and females. The primary outcome variable was disease-free survival at 1 year following definitive revision. Multivariable logistic regression analysis was performed to determine risk factors for failure. Of the 470 patients meeting final eligibility criteria, 250 were male and 226 were female (2 males and 4 females had a joint infection of either the contralateral side or a different joint and were treated as separate records). Of the patients in the cohort, 80% of the males (200/250) and 80% of the females (181/226) were found to be disease-free at 1-year follow-up (*p* > 0.99). Multivariable logistic regression analysis showed that nicotine use and diabetes, but not sex, were significant predictors of failure. Our study did not find a relationship between sex and outcome of revision for PJI. Further research is required to determine whether differences exist between males and females in the expression of PJI and outcomes following treatment.

## 1. Introduction

With an incidence of 1–3%, periprosthetic joint infection (PJI) is a rare but devastating complication following total joint arthroplasty (TJA) [1]. The gold standard treatment of chronic PJI in the United States is a two-stage exchange with the success rate ranging between 70 and 90% depending on host factors and infection characteristics [2,3,4,5,6,7]. When two-stage exchange fails to control or eradicate infection, subsequent treatment options are limited, and outcomes are dismal [8]. Current projections suggest that the number of primary total hip and knee arthroplasties in the United States will increase up to two-fold by the year 2030 [9]. As such, the number of two-stage exchanges performed due to chronic PJI is expected to increase as well. Understanding all risk factors for failure following two-stage exchange for PJI may provide an insight into preventing future failures.

Although the rate of primary TJA is greater in women, the rate of revision is higher in men [10]. Interestingly, several studies report that males have a greater risk for periprosthetic joint infection following primary TJA [11,12,13,14,15]. Recently, Fricka et al. showed males to be at a significantly greater risk of revision for infection following TKA [15]. Despite an understanding of sex and gender disparities in primary TJA, the literature lacks studies examining the relationship between sex and outcomes following single and two-stage exchange for PJI.

We present a study that examines whether disease-free survival at the 1-year follow-up after one- and two-stage revision for PJI differs between men and women. As a secondary aim, we compared the profiles of infecting organisms and comorbidity burdens between the sexes.

## 2. Results

Of the 470 patients, 250 were male and 226 were female (2 males and 4 females had a joint infection of either the contralateral side or a different joint and were treated as separate records). The characteristics of the sex groups were similar. There were no statistically significant differences noted in age, BMI, nicotine use, diabetes status, malnutrition (defined by serum albumin <3.5 g/dL), or MSIS infection type, host type, or local extremity/wound grade between the two sexes (Table 1). Specifics of the procedures performed can be seen in Table 2.

The disease-free success rate at one-year follow-up (Table 3) was similar (*p* > 0.99) between men and women. A total of 80% of men (200 of 250) and 80% of women (181 of 226) were found to be disease-free at the 1 year follow-up.

The results of a multivariable logistic regression modeling the odds of failure indicated that after controlling for patient and infection characteristics, there was no significant association between patient sex and failure. Additionally, malnutrition and diabetes were independent risk factors for failure. There were no statistically significant associations between failure and age, BMI, smoking, or MSIS infection category (Table 4).

Methicillin-sensitive *Staph aureus* was the most common infecting organism in both males and females. Methicillin-resistant *Staph aureus*, *Strep agalactiae*, Methicillin-resistant *Staph epidermidis*, *E. coli*, other coagulase-negative *Staph* species, and other streptococcus species were amongst the top ten most prevalent infecting organisms for both sexes. *Staph lugdunensis*, *Enterococcus faecalis*, and *Cutibacterium acnes* were in the top ten most prevalent infecting organisms for males but not females while pseudomonas, other enterococcus species, and other Gram-negative rod species were in the top ten most prevalent infecting organisms for women but not men (Table 5). There was no significant association between the infecting organisms and the sex of the patient.

## 3. Discussion

As the number of TKA and THA procedures continues to rise, we can expect the number of revisions due to PJI to increase. Therefore, understanding the patient risk factors for complications following these procedures is important. Although studies reporting the differences in preoperative presentation, intraoperative variables, and postoperative outcomes between sexes following primary TKA and THA exist, the literature examining how sex affects outcomes following procedures for PJI is lacking [16,17,18,19,20,21,22,23,24,25,26,27]. In our study, we sought to determine whether sex affects outcomes following single or two-stage exchange for PJI and what potential differences between the populations may explain this. 

During the time frame of January 2010–January 2021 at our institution, more female patients underwent a primary TJA than males, although more males went on to require a revision for PJI. This phenomenon has been well reported in the literature [11,13,14]. However, the association of patient sex on the outcomes following these revision procedures is not currently known. In our study, we found that sex was not significantly associated with re-revision at 1 year following revision for PJI. PJI eradication rates were identical for men and women, at 80%.

A meta-analysis performed by Kong et al. found that male sex, age, obesity, alcohol use, diabetes mellitus, urinary tract infections, and rheumatoid arthritis were all associated with periprosthetic joint infection [12]. We found that more males underwent two-stage revision, which is in accordance with this study. In our cohort, there were no significant differences between males and females regarding demographics (including age, BMI, ethnicity, and race), diabetes, nicotine use, malnutrition, MSIS infection type, host grade, or local extremity/wound grade, or organism profile. The discrepancy between the sexes as it pertains to revision surgery for PJI may be due to a factor not captured by our study. Detailed medical histories were not reported in our study, so it is possible that differences in medical comorbidities existed between cohorts that were not captured by the McPherson classification system. Additionally, we did not obtain social historical data such as ethanol consumption, which may also be different between males and females in the cohort.

Basques et al. found that following primary TJA, males had higher rates of postoperative mortality, surgical site infection, and shorter hospital stays while females had an overall higher adverse event rate, including rates of UTI, thromboembolic events, and requirement of blood transfusions [14]. Relevant to our study is the increased rate of SSIs in males given that SSI is a known risk factor for the development of PJI. Risk factors for SSI have been identified and include the male sex, younger age, current/former smoking, diabetes, obesity, operative time, wound dehiscence, and ASA > 2 [28,29]. 

There is a precedent for differences in infection susceptibility between males and females in other systems. It has been well reported that females are at an increased risk for UTI relative to males [30]. The strongest risk factors for UTI are previous UTI and insulin-treated diabetes while female sex, obesity, and genetic susceptibility have shown to be minor risk factors [31,32]. UTIs following TJA are most commonly catheter associated and UTI is a leading cause of sepsis following surgery [33]. 

Although we identified seven organisms amongst the ten most prevalent infecting organisms in both sexes, no statistically significant differences in prevalence of these organisms existed between the sexes. We identified three organisms amongst the ten most prevalent infecting organisms in males only, and three amongst the ten most prevalent in females only. Although females are at an increased risk for UTI and *E. coli* remains the most common causative agent for both uncomplicated and complicated UTI, we did not find a statistically significant difference between prevalence rates of *E. coli* between the sexes (*p* = 0.39) [32]. Our study may lack the power necessary to detect significant differences in the prevalence of infecting organisms between the sexes. Future work is required to better understand the differences in infecting organism profiles between the sexes and how this may affect outcomes following revision for PJI.

### Limitations

Our study has several limitations. The first is the relatively small population sampled and potential lack of power required to detect important differences between the sexes. As previously noted, we did not include granular details of each patient’s past medical and social histories, which may contain important elements that affect outcomes following single and two-stage exchange for PJI. Our study population consists largely of referred patients so the specific ratio of male vs. female patients treated with primary TKAs and THAs in the referring regions is unknown and may have introduced a selection bias. Finally, our study was retrospective in nature, which inherently carries many biases. 

## 4. Materials and Methods

After institutional review board approval, we queried our institutional periprosthetic joint infection registry to identify all patients with a Musculoskeletal Infection Society (MSIS) confirmed PJI indicated for a single or two-stage exchange from January 2010 to January 2021. Furthermore, patients were eligible for inclusion in the retrospective cohort study if they were at least 18 years old, had a known infecting organism or confirmed culture-negative infection, and underwent the first stage resection as part of a planned single or two-stage revision arthroplasty. Patients were excluded if treatment details were unavailable in the PJI registry; if surgical intervention occurred prior to January 2010 or after January 2021; if the original spacer or single-stage procedure was for primary septic arthritis; if surgical treatment was a planned definitive spacer insertion with no planned reimplantation; or, if the patient was indicated for a planned two-stage fusion.

A retrospective review of the registry data and the electronic health records was conducted to record demographic variables (age, sex, body mass index (BMI), race, ethnicity, comorbidities, and medical history), preoperative and intraoperative microbiology, serology [Erythrocyte Sedimentation Rate (ESR) and C-reactive protein (CRP)], and pathology. Incidence of primary and aseptic revision arthroplasty were also included and stratified between the sexes. Success was defined as disease-free survivorship (defined as Fillingham criteria 1A or 1B outcomes) at 1 year following reimplantation (either 1 year from one-stage procedure or 1 year from reimplantation in two-stage procedures) [34].

All two-stage procedures employed the use of a high dose antibiotic laden cement spacer, constructed using Palacos bone cement. For each pack of cement, we added 2 g of vancomycin and 2.4 g of tobramycin to create the spacer. The number of packs used was determined on a case-by-case basis and was left to the treating surgeon’s discretion based on desired fixation and bone defects encountered during surgery. Bone defects were not routinely described in operative reports so were out of the scope of this retrospective study. In one-stage procedures, as expected, no temporary spacer was used. In all cases (one- and two-stage exchange) patients received IV antibiotics via PICC line for six weeks. The choice of antibiotic was determined by operative cultures and in collaboration with infectious disease consultation. In one-stage procedures, patients received an additional minimum six months of appropriate oral antibiotic. In two-stage procedures, after the six weeks of IV antibiotics, the patients went through a drug holiday at which point the patient underwent a complete infectious workup to determine candidacy for reimplantation. The criteria for reimplantation included negative cultures on joint aspiration, down trending ESR/CRP, healed wound with no erythema, and no clinical concern for ongoing infection. Cell count and differential from aspiration were interpreted by the treating surgeon in conjunction with all clinical available information. After second stage reimplantation, the patients received a minimum of six months of oral antibiotic. Implant type, mode of fixation, and use of cement was determined on a case-by-case basis according to the treating surgeon.

All data were organized using a centralized database program (Research Electronic Database Capture [REDCap], Nashville, TN, USA) and underwent statistical analyses using SAS version 9.4 (SAS Institute, Cary, NC, USA; http://www.sas.com/software/sas9 accessed on 22 May 2022). Standard univariate descriptive statistics were used including frequency and proportion as well as measures of central tendency. Bivariate statistical associations between categorical variables were determined using Chi-Square Tests. The distributions of all continuous data were assessed using visual methods, e.g., quantile–quantile plots and histograms as well as the statistical tests Kolomogorov–Smirnov and Shapiro–Wilk statistical tests. For normally distributed data, means and standard deviations were calculated and Independent T-Tests were used to assess bivariate statistical associations. For data that were not normally distributed, median and interquartile range (IQR) were calculated and Wilcoxon Tests were used to assess bivariate statistical associations. Finally, a multivariable logistic model was built to model the odds (and 95% confidence interval) of failure for females compared to males (referent category) after adjusting for the statistical effects of age, BMI, nicotine use, presence of diabetes, nutritional status, and MSIS infection type.

### Study Sample

**Figure 1:**
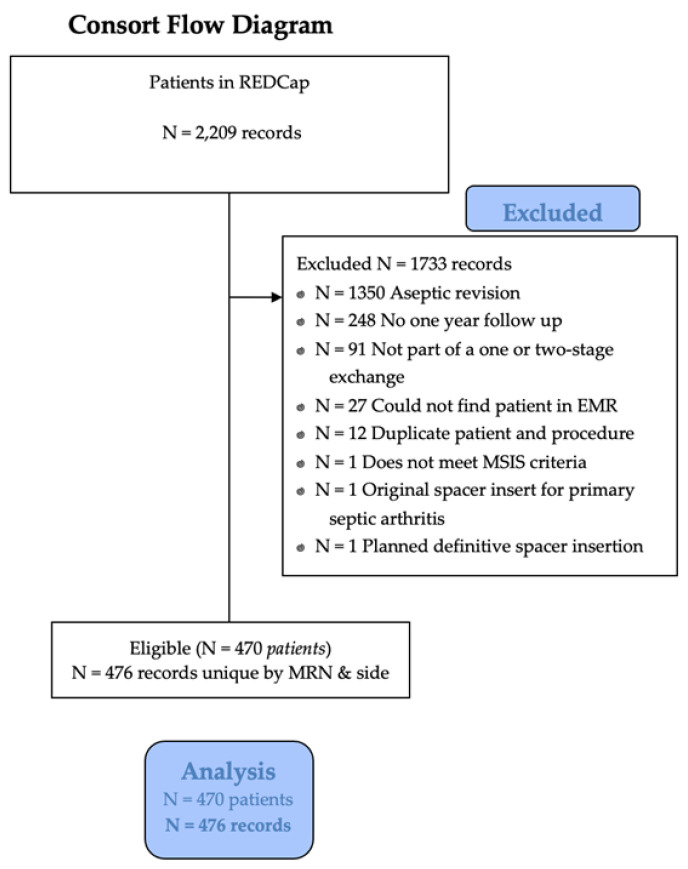
Consort diagram.

At a single private practice institution, 14,782 patients [6973 (47.2%) males and 7809 (52.8%) females] underwent a primary TJA during the study time frame. A total of 6652 patients [3036 (45.6%) males and 3616 (54.4%) females] underwent a revision TJA during the study time frame. Of these 6652 patients, 2209 patients [1155 (52.3%) males and 1054 (47.7%) females] underwent a revision TJA for PJI during the study time frame. The final cohort included 470 patients (476 procedures) that met final eligibility criteria for inclusion in the study and were included in the analysis. A consort diagram describing the selection of the patients in this study is seen in Figure 1.

## 5. Conclusions

Our study lends evidence that supports the assertion that males are treated with revision for PJI more commonly than females. However, sex did not affect the ultimate outcome of surgery, and we were unable to detect characteristics in males and females that differentiated the two populations. Further research is required to determine why males are more susceptible to PJI than females yet seem to be equally capable of clearing infection after revision for PJI. As details are elucidated regarding the specific mechanism for this discrepancy, preventative strategies can be implemented to better optimize patients for these operations.

## Figures and Tables

**Table 1:** Demographics.

	Male (N = 248)	Female (N = 222)	Overall (N = 470)	*p*-Value
Age, mean (SD)	65.1 (10.4)	66 (9.3)	65.5 (9.9)	0.365
BMI, median (IQR)	30.4 (26.8, 35)	31.3 (25.7, 37.1)	30.7 (26.4, 36)	0.623
Ethnicity, n (%)				
Not Hispanic or Latino	241 (97.2%)	212 (95.5%)	453 (96.4%)	0.999
Hispanic or Latino	3 (1.2%)	3 (1.4%)	6 (1.3%)	
Missing	4 (1.6%)	7 (3.2%)	11 (2.3%)	
Race, n (%)				
White	215 (86.7%)	177 (79.7%)	392 (83.4%)	0.216
Black, African American	31 (12.5%)	41 (18.5%)	72 (15.3%)	
American Indian, Alaska Native	1 (0.4%)	1 (0.5%)	2 (0.4%)	
Asian	1 (0.4%)	2 (0.9%)	3 (0.6%)	
Missing	0 (0%)	1 (0.5%)	1 (0.2%)	
Nicotine				
No	210 (84.7%)	187 (84.2%)	397 (84.5%)	0.897
Yes	36 (14.5%)	34 (15.3%)	70 (14.9%)	
Missing	2 (0.8%)	1 (0.5%)	3 (0.6%)	
Diabetes				
No	174 (70.2%)	171 (77.0%)	345 (73.4%)	0.064
Yes	74 (29.8%)	49 (22.1%)	123 (26.2%)	
Missing	0 (0%)	2 (0.9%)	2 (0.4%)	
Malnutrition				
No	197 (79.4%)	180 (81.1%)	377 (80.2%)	0.81
Yes	46 (18.5%)	39 (17.6%)	85 (18.1%)	
Missing	5 (2.0%)	3 (1.4%)	8 (1.7%)	
MSIS Infection Type				
III: Late chronic infection (>4 weeks duration)	218 (87.9%)	185 (83.3%)	403 (85.7%)	0.503
II: Hematogenous infection (<4 weeks duration)	23 (9.3%)	27 (12.2%)	50 (10.6%)	
I: Early postoperative infection (<4 weeks postoperative)	5 (2.0%)	6 (2.7%)	11 (2.3%)	
Missing	2 (0.8%)	4 (1.8%)	6 (1.3%)	
MSIS Infection Type Combined				
III: Late chronic	218 (87.9%)	185 (83.3%)	403 (85.7%)	0.271
I and II combined	28 (11.3%)	33 (14.9%)	61 (13.0%)	
Missing	2 (0.8%)	4 (1.8%)	6 (1.3%)	

**Table 2:** Procedures.

	Male (N = 250)	Female (N = 226)	Overall (N = 476)	*p*-Values
Infection Procedure				
1 Stage	26 (10.4)	21 (9.3)	47 (9.9)	0.6857
2 Stage	224 (89.6)	205 (90.7)	429 (90.1)	
Procedure				
Revision THA	86 (34.4)	90 (39.8)	176 (37.0)	0.221
Revision TKA	164 (65.6)	136 (60.2)	300 (63.0)	

**Table 3:** One-year disease-free success rates.

	Male (N = 250)	Female (N = 226)	Overall (N = 476)	*p*-Value
Yes	200 (80.0%)	181 (80.1%)	381 (80.0%)	
				>0.99
No	50 (20.0%)	45 (19.9%)	95 (20.0%)	

**Table 4:** Multivariable logistic regression modeling the odds of failure.

Effect	Failure OR (95% CI)	*p*-Value
Sex (Female vs. Male)	1.06 (0.652, 1.735)	0.8045
Age at Surgery	0.98 (0.955, 1.007)	0.1429
BMI	0.998 (0.961, 1.036)	0.9029
Nicotine (No vs. Yes)	0.565 (0.293, 1.091)	0.0892
Diabetes (No vs. Yes)	0.538 (0.311, 0.929)	0.0261
Malnutrition (No vs. Yes)	0.351 (0.2, 0.615)	0.0003
MSIS Infection (Combined I and II vs. III: Late Chronic)	1.29 (0.656, 2.537)	0.4607

**Table 5:** Most prevalent infecting organisms by sex.

Male	Count	Female	Count
Methicillin-Sensitive *Staph a*	90 (19.69%)	Methicillin-Sensitive *Staph a*	67 (16.63%)
Other Coagulase Negative *Staph*	78 (17.07%)	Methicillin-Resistant *Staph a*	63 (15.63%)
Methicillin-Resistant *Staph a*	67 (14.66%)	Other Coagulase Negative *Staph*	56 (13.90%)
*Strep agalactiae*	19 (4.16%)	Other *Strep*	18 (4.47%)
*Staph lugdunensis*	18 (3.94%)	Methicillin-Resistant *Staph epidermidis*	17 (4.22%)
Other *Strep*	17 (3.72%)	*Strep agalactiae*	17 (4.22%)
*Enterococcus faecalis*	15 (3.28%)	*Escherichia coli*	16 (3.97%)
Methicillin-Resistant *Staph epidermidis*	15 (3.28%)	*Pseudomonas*	14 (3.47%)
*Cutibacterium acnes*	14 (3.06%)	Other *Enterococcus*	10 (2.48%)
*Escherichia coli*	13 (2.84%)	Other Gram-Positive Rod	10 (2.48%)
Other	111 (24.29%)	Other	115 (28.54%)

## Data Availability

The data presented in this study are available on request from the corresponding author. The data are not publicly available due to privacy restrictions.
